# *Drosophila melanogaster* pigmentation demonstrates adaptive phenotypic parallelism over multiple spatiotemporal scales

**DOI:** 10.1093/evlett/qraf008

**Published:** 2025-04-08

**Authors:** Skyler Berardi, Jessica A Rhodes, Mary Catherine Berner, Sharon I Greenblum, Mark C Bitter, Emily L Behrman, Nicolas J Betancourt, Alan O Bergland, Dmitri A Petrov, Subhash Rajpurohit, Paul Schmidt

**Affiliations:** Department of Biology, University of Pennsylvania, Philadelphia, PA, United States; Department of Biology, Stanford University, Stanford, CA, United States; Department of Biology, University of Pennsylvania, Philadelphia, PA, United States; Department of Biology, Stanford University, Stanford, CA, United States; DOE Joint Genome Institute, Lawrence Berkeley National Laboratory, Berkeley, CA, United States; Department of Biology, Stanford University, Stanford, CA, United States; Department of Biology, University of Pennsylvania, Philadelphia, PA, United States; Department of Biological Sciences, Dartmouth College, Hanover, NH, United States; Department of Biology, University of Pennsylvania, Philadelphia, PA, United States; Department of Biology, Stanford University, Stanford, CA, United States; Department of Biology, University of Virginia, Charlottesville, VA, United States; Department of Biology, Stanford University, Stanford, CA, United States; Department of Biology, University of Pennsylvania, Philadelphia, PA, United States; Biological and Life Sciences Division, School of Arts and Sciences, Ahmedabad University, Ahmedabad, Gujarat, India; Department of Biology, University of Pennsylvania, Philadelphia, PA, United States

**Keywords:** adaptation, parallel evolution, evolutionary genomics, insects

## Abstract

Populations are capable of responding to environmental change over ecological timescales via adaptive tracking. However, the translation from patterns of allele frequency change to rapid adaptation of complex traits remains unresolved. We used abdominal pigmentation in *Drosophila melanogaster* as a model phenotype to address the nature, genetic architecture, and repeatability of rapid adaptation in the field. We show that *D. melanogaster* pigmentation evolves as a highly parallel and deterministic response to shared environmental variation across latitude and season in natural North American populations. We then experimentally evolved replicate, genetically diverse fly populations in field mesocosms to remove any confounding effects of demography and/or cryptic structure that may drive patterns in wild populations; we show that pigmentation rapidly responds, in parallel, in fewer than 15 generations. Thus, pigmentation evolves concordantly in response to spatial and temporal climatic axes. We next examined whether phenotypic differentiation was associated with allele frequency change at loci with established links to genetic variance in pigmentation in natural populations. We found that across all spatial and temporal scales, phenotypic patterns were associated with variation at pigmentation-related loci, and the sets of genes we identified at each scale were largely nonoverlapping. Therefore, our findings suggest that parallel phenotypic evolution is associated with distinct components of the polygenic architecture shifting across each environmental axis to produce redundant adaptive patterns.

## Teaser

Defining the predictability and pace of adaptation in wild populations are key aims of evolutionary biology, and we explored these dynamics using *Drosophila melanogaster* abdominal pigmentation as a model phenotype. We sought to understand whether pigmentation patterns vary in a consistent manner across three independent spatiotemporal axes: wild populations sampled over a latitudinal cline, wild populations sampled across seasons, and experimental populations sampled across seasons in field mesocosms. We measured abdominal melanization patterns following common garden treatment and found that pigmentation evolves as a parallel response across all scales, in which reduced melanization is associated with warmer temperatures. These patterns were also associated with variation in known pigmentation genes. The parallel shifts in pigmentation across shared environmental axes provide strong evidence that this complex trait is evolving adaptively; thus, we demonstrate for the first time that pigmentation adapts predictably to environmental variation over ecological timescales.

Characterizing the dynamics of adaptation in the field is a central goal of evolutionary biology. To better understand how populations may respond to environmental change, it is necessary to define the tempo and repeatability of evolution at both the phenotypic and genomic levels. These questions have been explored across multiple scales, from macroevolutionary patterns and broad adaptive radiations (Knoll & Carroll, 1999) to controlled laboratory experiments detailing microbial evolution (Lenski et al., 1991). Examining geographic clines in phenotypic and genotypic variation represents a classic approach to studying adaptation (Allen, 1877; Endler, 1977). Yet, both the exact timescale over which local adaptation occurs, and the respective roles of selection and demography in maintaining clines, are often obscured. Therefore, to understand how populations respond to the environment over ecological timescales, recent studies have leveraged high-resolution temporal sampling as a powerful approach to decode genetic changes that may contribute to adaptive evolution in the field (Barrett et al., 2008, 2019; Bitter et al., 2024; Enbody et al., 2023; Jones et al., 2018; Nosil et al., 2018; Roberts Kingman et al., 2021; Rudman et al., 2022). However, rarely have these approaches been combined to examine adaptation across multiple scales, and integrating spatial and temporal patterns provides an opportunity to address several fundamental questions about the dynamics of adaptation in natural populations. In particular, we can test whether patterns of adaptive change are parallel across multiple environmental axes that arise over space and time, define the timescale over which adaptive patterns can be established, and examine how allele frequencies for putatively causal loci covary with phenotypic patterns. Therefore, to explore the pace and predictability of adaptation in the field and integrate phenotypic patterns with genomic shifts, we studied the evolution of a complex trait across multiple spatiotemporal scales in *Drosophila melanogaster.*

*Drosophila* species have been a classic model for exploring evolution over ecological timescales (e.g., Dobzhansky, 1943). *D. melanogaster* exhibits signatures of rapid evolution across a broad array of fitness-related phenotypes (Behrman et al., 2015, 2018; Erickson et al., 2020; Rajpurohit et al., 2017, 2018; Schmidt & Conde, 2006). Additionally, seasonally cycling polymorphisms have been detected in populations of *D. melanogaster*, representing genomic signatures of fluctuating selection (Bergland et al., 2014; Bitter et al., 2024; Machado et al., 2021; Nunez et al., 2024; Rudman et al., 2022), and studies have begun to integrate rapid phenotypic adaptation with underlying patterns of genetic variation in this system (Erickson et al., 2020; Rajpurohit et al., 2018). We thus aimed to probe for adaptive parallelism across multiple scales using a complex, fitness-related trait with a well-characterized genetic basis. Thousands of polymorphisms vary latitudinally and seasonally in *D. melanogaster* (Bergland et al., 2014; Machado et al., 2021; Rudman et al., 2022), so studying a trait with a known genetic basis would enable our analyses to target variation with established links to phenotype. We selected *D. melanogaster* pigmentation as our model trait, and our motivation for studying pigmentation was threefold: it exhibits extensive phenotypic variation in wild populations (Kronforst et al., 2012), it is putatively adaptive (Bastide et al., 2014; Pool & Aquadro, 2007), and it has a well-studied genetic architecture (Bastide et al., 2013; Dembeck et al., 2015; Lafuente et al., 2024).

*Drosophila* pigmentation varies dramatically in natural environments both across and within species (Kronforst et al., 2012). Latitudinal clines for *D. melanogaster* pigmentation have been described in Europe, India, and Australia, in which melanization increases with latitude (Das, 1995; David et al., 1985; Munjal et al., 1997; Parkash et al., 2008; Rajpurohit et al., 2008a; Telonis-Scott et al., 2011). Additionally, pigmentation has been shown to increase with altitude in Sub-Saharan Africa and India (Bastide et al., 2014; Dev et al., 2013; Munjal et al., 1997; Parkash et al., 2008; Pool & Aquadro, 2007; Rajpurohit et al., 2008a). These repeated clinal patterns suggest pigmentation is likely an adaptive response to environmental conditions that vary predictably over latitudinal and altitudinal gradients, such as temperature, which is a hypothesized driver of pigmentation based on evidence that melanin aids in thermoregulation (Freoa et al., 2023). Crucially, the pathways underlying melanin and sclerotin biosynthesis in each abdominal tergite are well-characterized (Kopp et al., 2003; True, 2003; Wittkopp et al., 2003). Melanization is influenced by major-effect genes including *tan*, *ebony*, *yellow*, *bab1*, and *bab2* (Couderc et al., 2002; Kopp et al., 2000; True et al., 2005; Wittkopp et al., 2002; Wright, 1987), and natural variation in pigmentation has been mapped to single nucleotide polymorphisms (SNPs) in both major- and minor-effect loci (Bastide et al., 2013; Dembeck et al., 2015; Endler et al., 2016; Lafuente et al., 2024). Therefore, given that segregating variants for canonical pigmentation genes exist in natural populations of *D. melanogaster*, we hypothesized that adaptive shifts in pigmentation may be driven by repeated selection at common variants of major-effect loci, similar to alleles of *Mc1r* and *Agouti* driving adaptive pigmentation patterns in wild populations of mice (e.g., Barrett et al., 2019; Hoekstra et al., 2006; Manceau et al., 2011). However, the canonical *Drosophila* pigmentation genes are also highly pleiotropic (Massey & Wittkopp, 2016; Wittkopp & Beldade, 2009), which may constrain selection over ecological timescales (Paaby & Rockman, 2013). In this case, pigmentation patterns may not be driven by shifts in major-effect pigmentation genes, but instead by selection across variants that are less subject to pleiotropic constraints (Kostyun et al., 2019).

We studied *D. melanogaster* pigmentation in wild populations to test several fundamental hypotheses about the dynamics of adaptation. We sought to characterize whether patterns of phenotypic and genomic evolution are parallel across multiple scales, and to identify whether timescale modulates how patterns of genetic variation map to phenotype. We began by defining pigmentation patterns along three axes of spatiotemporal environmental variation. If variance in melanization is driven by selection, we anticipated that pigmentation would adapt as a predictable response across each environmental axis. We first examined melanization patterns in populations along the East Coast of North America, an uncharacterized latitudinal cline. However, because we are unable to resolve the timescale over which clinal patterns are established or the contributing influence of admixture (Bergland et al., 2016), we next asked whether pigmentation varies in response to seasonal change in a focal orchard. This allowed us to define the pace of evolution, and to ask whether seasonal and latitudinal patterns are concordant based on shared variation in environmental factors, such as temperature. Finally, we experimentally evolved *D. melanogaster* populations in field mesocosms, where we eliminated confounding effects of migration and/or cryptic population structure that could be present in the wild populations. By measuring seasonal pigmentation patterns in our replicated experimental populations, we directly defined the adaptive dynamics of melanization over ecological timescales. After establishing parallelism in the phenotypic response across scales, we then explored whether shifts in pigmentation genes consistently underpin observed phenotypic patterns. We aimed to narrow the genetic variation we examined to candidates with an established connection to pigmentation in natural populations, so we focused on polymorphisms in major- and minor-effect pigmentation genes that likely represent commonly segregating variants (Bastide et al., 2013; Dembeck et al., 2015). This enabled us to determine whether repeated phenotypic adaptation is associated with predictable variation in validated, pigmentation-related SNPs across each spatial and temporal scale examined.

## Methods

### Fly collection and maintenance

#### Wild orchard populations

*D. melanogaster* individuals were collected from orchard populations located along the East Coast (United States). Latitudinal populations were sampled in late spring across six sites: Lancaster, MA (42.45, −71.67); Media, PA (39.88, −75.41); Charlottesville, VA (38.00, −78.47); Athens, GA (33.95, −83.36); Jacksonville, FL (30.48, −81.70); and Homestead, FL (25.50, −80.48). Latitudinal collections are described in Paaby et al. (2010), Paaby et al. (2014), Bergland et al. (2014), Behrman et al. (2018), and Machado et al. (2021), and these samples have also been sequenced and incorporated into the *Drosophila* Evolution over Space and Time (DEST) dataset (Kapun et al., 2021). Additionally, seasonal collections were done in Media, PA, over 6 years (2010–2015) (Behrman & Schmidt, 2022), and were sequenced as poolseq data (Kapun et al., 2021; Machado et al., 2021). Early-season collections were made over the first week of June, and late-season collections were completed in early November (Behrman & Schmidt, 2022; Behrman et al., 2015; Rajpurohit & Schmidt, 2016). All flies were captured in the field by direct aspiration on windfall fruit, brought into the laboratory, and typed to species to establish *D. melanogaster* isofemale lines. These lines were then maintained at low density culture at 24 °C, 12L:12D photoperiod, on standard cornmeal–molasses–agar medium. After two generations of common garden treatment under these conditions in the lab, all F3 (third filial generation) individuals from a given isofemale line were preserved at 80% EtOH at −80 °C until analysis.

#### Experimental orchard populations

On June 30, 2016, we founded each of 10 replicate, outdoor mesocosms with 500 males and 500 females from an outbred founder population that was established by recombining a panel of 80 inbred lines, as described by Rudman et al. (2022). Each 8 m^3^ mesocosm contains a dwarf peach tree and vegetative ground cover to provide a semi-natural environment for fly rearing. The experiment was conducted from June 30 to October 5, 2016, and each population was provided 1 L of cornmeal–molasses medium each week. To assess evolutionary patterns, *D. melanogaster* eggs were sampled from each mesocosm at three timepoints (August 5, September 5, and October 5). DNA was sequenced at all three timepoints, and flies were preserved for pigmentation measurements at two timepoints (August 5 and October 5). 100 females were pooled from each mesocosm to conduct whole genome sequencing (using the DNA extraction, sequencing, and analysis pipelines described in Rudman et al. (2022) and Bitter et al. (2024)). To measure melanization at each timepoint, flies sampled from each mesocosm were reared for two generations in laboratory, common garden conditions (25 °C, 12L:12D, ~30 eggs per vial), to control for any effects of developmental plasticity. Five-day old F3 individuals corresponding to each mesocosm and timepoint were stored in 80% EtOH at −80 °C for later phenotyping.

### Scoring fly samples for abdominal pigmentation

#### Wild orchard populations

We scored female abdominal pigmentation following common garden treatment using a lateral view of the abdomen, giving values from 0 (0% of the tergite is melanized) to 10 (100% melanized) for each of seven abdominal tergites as described by David et al. (1990). We only scored female flies, as abdominal pigmentation in male flies is less variable among individuals. Scoring was completed by multiple independent investigators, and these observations were quality checked by the first author. All population samples were assigned unique, anonymous identifiers and scored blindly of sample identity to avoid bias. Individual females were randomly selected from population samples stored at −80 °C and scored under a dissecting microscope; females were then discarded to avoid scoring the same individual twice. To measure latitudinal patterns, we scored all isofemale lines from each latitudinal population that had at least 10 individual females preserved at −80 °C. The number of isofemale lines with 10 females available to score across populations ranged from 17 to 30 lines (Supplementary Table S1A). To measure seasonal patterns, we sampled 10–20 isofemale lines from Media, PA, at early- and late-season timepoints, and scored 5–10 individuals per line (Supplementary Table S1B). We then calculated the mean pigmentation score for each isofemale line across seasons and years. Latitudinal pigmentation patterns were analyzed in R (v.4.4.0) with a linear mixed effects model using the package “nlme” (v.3.1.164) (Supplementary Table S2A). Here, latitude was modeled as a continuous fixed effect, and isofemale line was modeled as a random effect, according to the formula: lme(Pigmentation Scores ~ Latitude, random = ~1|Isofemale Line). Similarly for the seasonal (Media, PA) populations, we examined pigmentation patterns with a linear mixed effects model where year, season (early or late), and their interaction were modeled as fixed effects, and isofemale line was included as a random effect: lme(Pigmentation Scores ~ Year*Season, random = ~1|Isofemale Line) (Supplementary Table S2B).

#### Experimental orchard populations

Abdominal pigmentation of *D. melanogaster* females was scored using the rubric described above (David et al., 1990). The three most distal tergites were scored (#5–7), as they show the highest degree of variation. We first scored four replicates of 20 females sampled from our ancestral founder population. For the August 5 and October 5 field-evolved timepoints, 20 individuals from each mesocosm were randomly selected and scored from the F3 samples stored at −80 °C; we then calculated a mean pigmentation score for each mesocosm (Supplementary Table S1C). One of our initial 10 mesocosms was lost, so we scored patterns for the remaining nine mesocosms. We ran a linear mixed effects model in R (“nlme,” v.3.1.164) for pigmentation scores in each mesocosm between the summer and fall timepoints (Supplementary Table S2C). Here, timepoint was included as a fixed effect and was modeled as an ordinal variable, and mesocosm was included as a random effect nested within timepoint. We used the formula: lme(Pigmentation Scores ~ Timepoint, random = ~1|Mesocosm/Timepoint). We included scores from our mid- (August 5) and late-season (October 5) timepoints in this model. While we did score replicates from our ancestral founder to determine the mean baseline pigmentation levels, we did not include these replicates in our model because we did not score pigmentation for the exact subsets of founder flies that were used to seed each cage: so, we could not compare the pigmentation score of each individual cage at founding (June 30) to August 5 and October 5, only the general baseline.

### Genomic analyses of sequencing data

#### Selection of candidate SNPs

We based our candidate SNP selection on naturally segregating variants identified by two previous genome-wide association studies (GWAS) of abdominal pigmentation (Bastide et al., 2013; Dembeck et al., 2015). Our candidate list included polymorphisms in canonical genes of the biosynthetic pathway of sclerotin and melanin, as well as smaller-effect loci in genes outside of this core pathway, and it totaled 249 SNPs associated with ~110 genes (Supplementary Table S3). Bastide et al. (2013) collected wild samples in Austria and Italy, and Dembeck et al. (2015) used inbred lines of the Drosophila Genetic Reference Panel (DGRP). These GWAS focused on different tergites: Bastide et al. (2013) looked at pigmentation of the seventh tergite, and Dembeck et al. (2015) focused on the fifth and sixth and their relationship. Since our pigmentation scoring considered the fifth, sixth, and seventh tergites, we included all significant SNPs from both studies. We completed our analyses using SNP coordinates based on the *D. melanogaster* Release 5 (dm3) coordinate system, and coordinates of each SNP in both Release 5 and Release 6 have been provided (Supplementary Table S3).

#### Calculating a metric of latitudinal clinality of candidate SNPs

To measure the degree of clinality for each SNP, we performed a linear regression (“lm” in R) on SNPs’ frequencies across latitudes. Our metric for clinality was the absolute value of the regression coefficient of the resulting model. Latitudinal samples we analyzed were collected from Homestead, FL (2010), Atlanta, GA (2014), Charlottesville, VA (2014), Media, PA (2014), and Lancaster, MA (2014); however, we did not have genomic data available for Jacksonville, FL, to include in our analyses. Data for these samples were sourced from the DEST dataset (Kapun et al., 2021), and corresponding sample IDs were: “FL_ho_10_spring,” “GA_at_14_spring,” “VA_ch_14_spring,” “PA_li_14_spring,” and “MA_la_14_spring.” We excluded any SNPs that were present in less than three of the five samples.

#### Calculating a metric of seasonality of candidate SNPs in wild orchard populations

We determined that the allele frequency change from spring to fall and the repeatability of this shift were the key components of seasonality. Therefore, we considered periods where both a spring and a fall sample existed in a given year, and we calculated the magnitude of allele frequency change between seasons. We then averaged across these spring–fall transitions to get the yearly seasonal change. Seasonal samples were collected in Media, PA, from 2009 to 2015 (DEST dataset, Kapun et al., 2021), and corresponding sample IDs were: “PA_li_09_spring,” “PA_li_09_fall,” “PA_li_10_spring,” “PA_li_10_fall,” “PA_li_11_spring,” “PA_li_11_fall,” “PA_li_12_spring,” “PA_li_12_fall,” “PA_li_14_spring,” “PA_li_14_fall,” “PA_li_15_spring,” and “PA_li_15_fall.” Notably, we did not measure pigmentation patterns during the 2009 season, so this year is only represented in our genomic analyses; additionally, our genomic analyses did not include samples from 2013, which were included in our phenotypic analyses. We excluded SNPs with data in fewer than three out of a possible six spring–fall pairs.

#### Calculating a metric of seasonality of candidate SNPs in outdoor mesocosm populations

For experimental seasonal measurements, we performed a linear regression of allele frequencies across the three timepoints collected during 2016. While we only had pigmentation measurements available from August 5 and October 5 to include in our phenotypic analyses, DNA was sequenced from an additional timepoint (September 5), which was included in our genomic analysis. We designated the absolute value of the regression coefficient as the metric for seasonal change to identify SNPs that shifted in a large, concordant direction over time. We excluded SNPs with missing data in any of the samples, as there were only three timepoints.

#### Selection of matched control SNPs

We selected a group of at least 200 matched control SNPs for each candidate SNP to establish a null model of expected clinal and seasonal patterns. Control SNPs were located on the same chromosome further than 50 kb from the candidate SNP, found in the same type of region (exon, 5′UTR/3′UTR, intron, gene, or intergenic, in order of priority), and had the same relationship to the six major inversions that occur in wild North American populations (within an inversion, within 500 kb of a breakpoint, or further than 500 kb). After matching on the above characteristics, SNPs with an average frequency across samples within 0.01 of the average frequency of the candidate SNP were chosen as the control SNP pool for that candidate SNP. If fewer than 200 SNPs were in the pool for a candidate SNP, we increased the possible difference in frequency until the pool reached at least 200 SNPs. However, no control SNP had a frequency difference of more than 0.07 from its candidate SNP.

#### Tests of overall clinality and seasonality of the entire set of candidate SNPs

To determine the clinality and seasonality of candidate SNP frequency changes as a group, we leveraged matched control SNPs to create 1,000 bootstrap-resampled groups equal in size to the number of candidate SNPs that passed the minimum threshold for each metric. Each matched group included one control SNP drawn randomly from the pool of each candidate SNP with sufficient data. The averages of the clinal or seasonal metrics within each of the 1,000 matched groups provided a null distribution of 1,000 values, which was used to calculate the *p*-value of the average of the candidate group. By creating these bootstrap resamples that matched the properties of the original candidate SNPs, we determined if the candidate SNPs as a group were more likely to follow clinal and seasonal patterns observed in the pigmentation phenotype, given enrichment for a significant magnitude of absolute allele frequency change.

#### Tests of clinality and seasonality of individual candidate SNPs

To test if each individual SNP varied more latitudinally or seasonally than expected, we drew a sample of 100 matched controls from its pool, and we repeated this 100 times. For each selection of 100 control SNPs, we determined the proportion of controls with a metric greater than the candidate SNP. We considered a SNP significant if it was within the top 10% of matched controls in at least 90 trials and within the top 5% in at least half of the 100 trials, providing a significance cutoff of α = 0.05 ± 0.05.

#### Data analysis and figures

**Figure 1.**
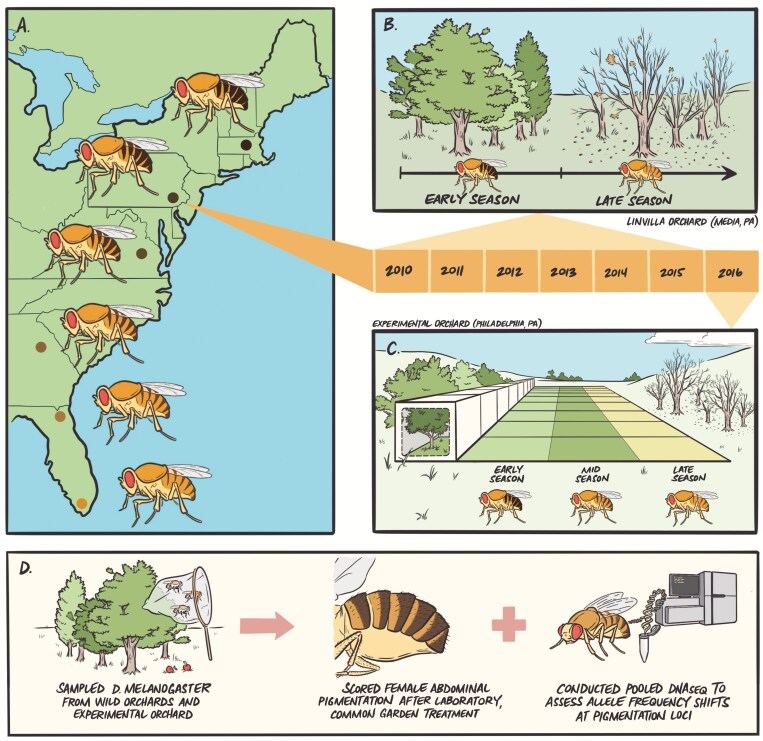
Experimental overview. (A) We sampled flies from six wild orchard populations ranging from Homestead, FL, to Lancaster, MA, and established isofemale lines in the laboratory. (B) We returned to a focal orchard in Media, PA, at early- and late-season timepoints and collected flies to capture evolutionary patterns following winter and summer conditions. (C) We then seeded outdoor mesocosms (*N* = 9) with an outbred population originating from early-season collections in Media, PA, and sampled flies at the end of summer (mid-season) and fall (late-season) to determine if seasonal patterns are recapitulated in experimental populations controlled for migration, drift, and cryptic population structure. (D) Across each wild or experimental context, we sampled flies, established lines in the lab, completed common garden treatment to remove environmental effects, and scored females for abdominal pigmentation. We also conducted pooled DNA sequencing on additional flies sampled from each population to map genomic patterns for candidate pigmentation SNPs.

All analyses were conducted in R (v.4.4.0) using the packages tidyverse (v.2.2.0), reshape2 (v.1.1.4), readxl (v.1.4.3), and nlme (v.3.1.164). Figures were made using Keynote (v.8.1) and R (v.4.4.0) using the packages ggplot2 (v.3.5.1), ggpubr (v.0.6.0), emmeans (v.1.10.2), and plotrix (v.3.8.4). Figure 1 was illustrated by Dr. Rush Dhillon (https://www.rushstudio.ca/).

## Results

### Abdominal pigmentation increases with latitude in North America

**Figure 2.**
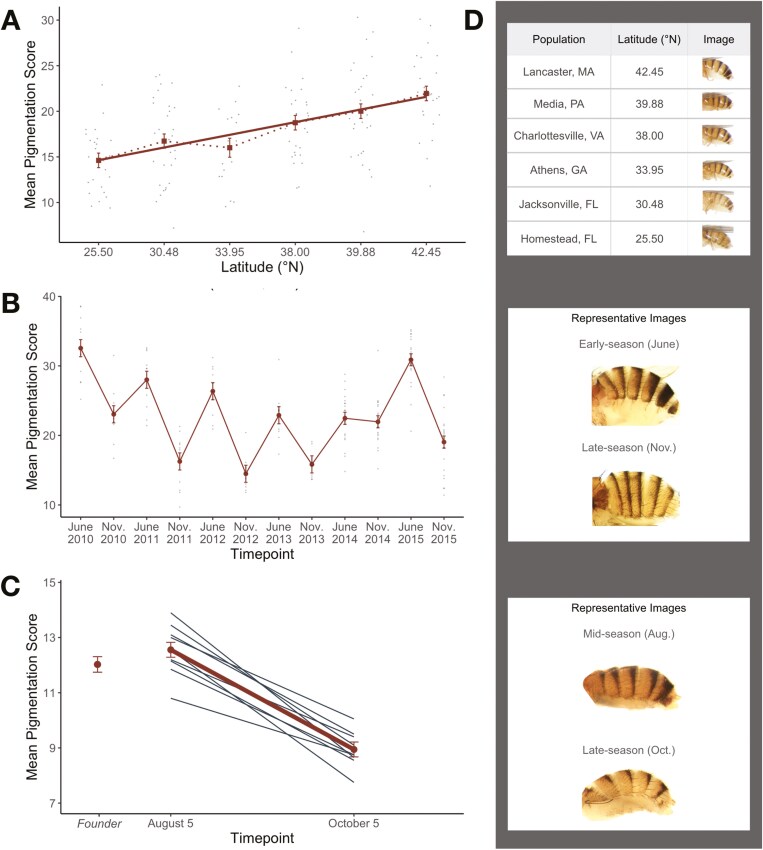
Phenotypic patterns and representative images for *D. melanogaster* populations in each natural context. (A) *D. melanogaster* pigmentation increases significantly with latitude. (B) A population of flies sampled from Media, PA, exhibit predictable and significant decreases in melanization from June to November over 6 years. (C) Decreases in pigmentation over seasonal time are recapitulated in semi-natural mesocosms (*N* = 9) located in Philadelphia, PA, and female flies adapt to exhibit significantly lighter pigmentation between August 5th and October 5th. The average pigmentation score of our spring-derived ancestral population (“Founder”) is also plotted. In all plots, mean pigmentation score and standard error are depicted alongside either points representing isofemale line means (A, B) or individual mesocosm means (C). (D) Representative images corresponding roughly to the mean of each population (from top to bottom): across latitudes, across seasons in wild populations, and across seasons in experimental populations. See the rubric designed by David et al. (1990) for a range of pigmentation score images.

We first determined whether *D. melanogaster* pigmentation varies latitudinally in North America. We predicted melanization would increase with latitude, consistent with documented latitudinal clines for abdominal pigmentation in *D. melanogaster* on other continents (Das, 1995; Munjal et al., 1997; Parkash et al., 2008; Rajpurohit et al., 2008a; Telonis-Scott et al., 2011). We measured female abdominal pigmentation for six populations along the East Coast of the United States, and all populations were phenotyped under common garden conditions to identify shifts in pigmentation driven by genetic changes (see “Methods”; Figure 1A and D; Supplementary Table S1A). Our results aligned with previously established clines: melanization increased with latitude in North America (*F*_5,157_ = 11.17, *p* < 0.0001; Figure 2A and D; Supplementary Table S2A). These parallel and independent clines across continents suggest pigmentation evolves adaptively in response to environmental conditions that vary predictably with latitude (Endler, 1977).

### Pigmentation varies predictably over seasonal timeframes in a wild orchard population

We next explored the temporal dynamics of melanization by examining flies collected across seasons in 6 successive years (Figure 1B and D; Supplementary Table S1B). We found that *D. melanogaster* females evolve seasonally and become significantly less melanized in the fall relative to the spring (*F*_1,148_ = 174.43, *p* < 0.0001; Figure 2B and D; Supplementary Table S2B), and this seasonal pattern was reset and repeated across the 6 years recorded. The magnitude of pigmentation change varied with year (*F*_5,148_ = 11.52, *p < *0.0001), with 2014 showing a dampened response. Thus, abdominal pigmentation evolves predictably and in parallel over the ~15 generations that span spring to fall in this species (Behrman & Schmidt, 2022). Additionally, the directionality of seasonal change is consistent with latitudinal patterns: early-season flies (“winter-evolved”) exhibited darker pigmentation, similar to populations sampled from higher latitudes. In contrast, flies were less melanized following the summer, consistent with populations originating from lower latitudes. This finding supports the hypothesis that environmental factors that vary in parallel over space and time (e.g., temperature) may be the primary drivers of phenotypic shifts.

### Replicate experimental populations of *D. melanogaster* exhibit highly rapid and parallel adaptation of pigmentation in field mesocosms

Finally, we characterized seasonal pigmentation patterns in replicate experimental populations (Figure 1C; Supplementary Table S1C), which enabled us to isolate the role of adaptation in driving phenotypic evolution while maintaining a semi-natural environment (Grainger et al., 2021; Rajpurohit et al., 2017, 2018; Rudman et al., 2019, 2022). We found late-season flies were less melanized relative to early-season flies across replicate mesocosms (*F*_1,8_ = 111.26, *p* < 0.0001; Figure 2C and D; Supplementary Table S2C), showing a concordant magnitude and directionality of phenotypic change to the wild populations. These parallel phenotypic patterns were observed across nine independent, replicate populations, strongly supporting our hypothesis that the observed shifts in melanization are adaptive. Together, our findings indicate that pigmentation exhibits a strikingly parallel phenotypic response to environmental conditions that vary across all spatiotemporal scales examined, and this represents the first demonstration that *D. melanogaster* pigmentation adapts rapidly in response to seasonally changing environments.

### Candidate pigmentation SNPs do not show enrichment for allele frequency change

**Figure 3.**
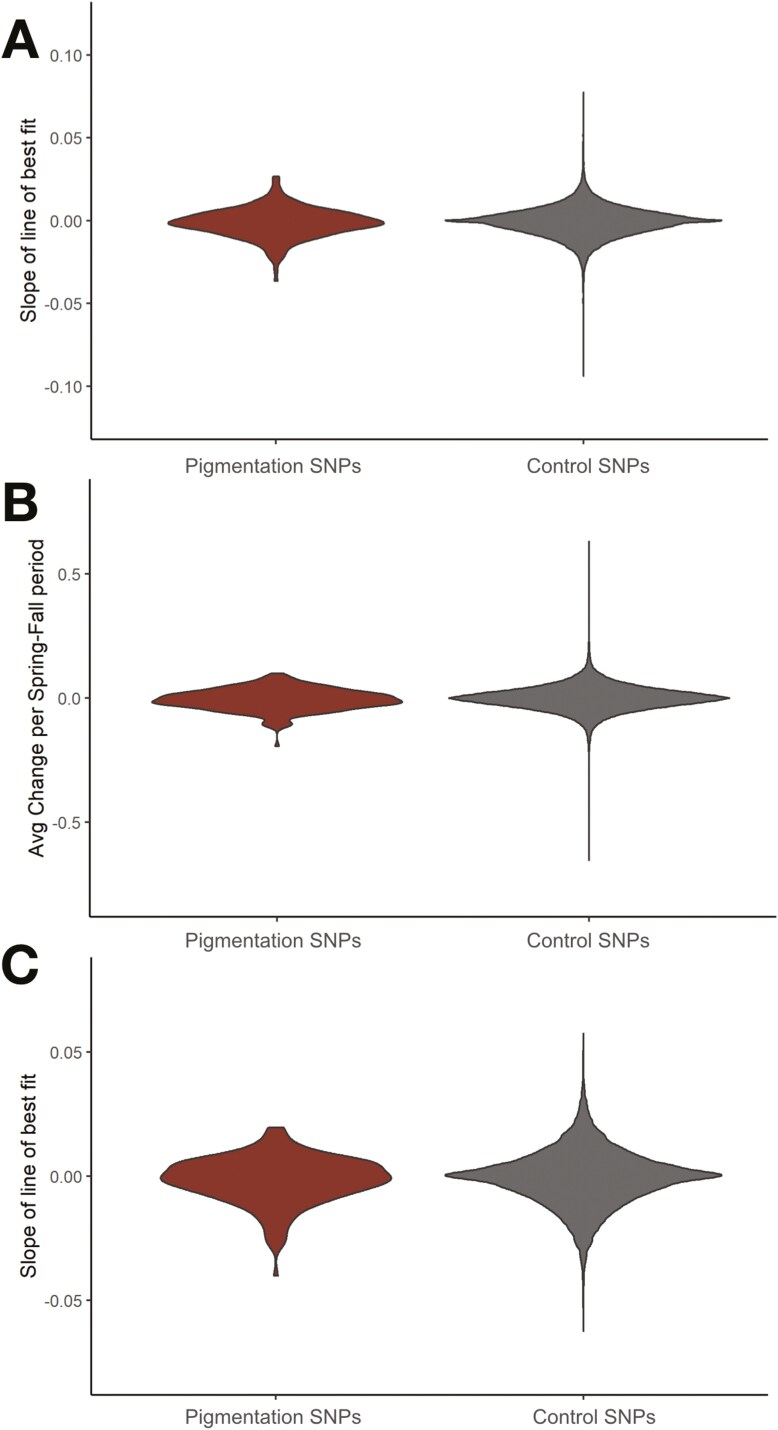
Distributions of candidate pigmentation SNPs vs. bootstrap resamples of matched control SNPs over space and time. Candidate pigmentation SNPs are not enriched as a group relative to matched controls across (A) latitudinal, (B) seasonal, or (C) experimental populations. Candidate SNPs were selected based on associations with pigmentation from GWAS of natural populations, and enrichment of candidate pigmentation SNPs was determined relative to groups of matched, control SNPs.

Defining these adaptive phenotypic patterns set the stage for us to characterize the underlying genomic architecture in each spatiotemporal context. We first asked whether our candidate pigmentation SNPs (Supplementary Table S3) were enriched as a group for latitudinal allele frequency variation relative to the genomic average. However, we determined that our candidate SNPs were not significantly more clinal relative to matched controls (asymptotic two-sample Kolmogorov–Smirnov test, *p* = 0.5233; Figure 3; Supplementary Table S4). We then repeated these analyses for our wild (*p* = 0.5174) and experimental (*p* = 0.8231) seasonal populations in the years corresponding to our phenotypic data, and similarly found that pigmentation SNPs were not enriched as a group for seasonal allele frequency change above the expectation by chance (Figure 3; Supplementary Table S4). These results were not entirely surprising, as it is possible *D. melanogaster* pigmentation patterns are being driven by selection across few loci, similar to other species (Hoekstra et al., 2006; Miller et al., 2007). Therefore, we next analyzed allele frequency shifts in each individual pigmentation SNP across all spatiotemporal scales.

### Individual pigmentation SNPs show significant allele frequency shifts across latitudinal, seasonal, and experimental populations

**Figure 4.**
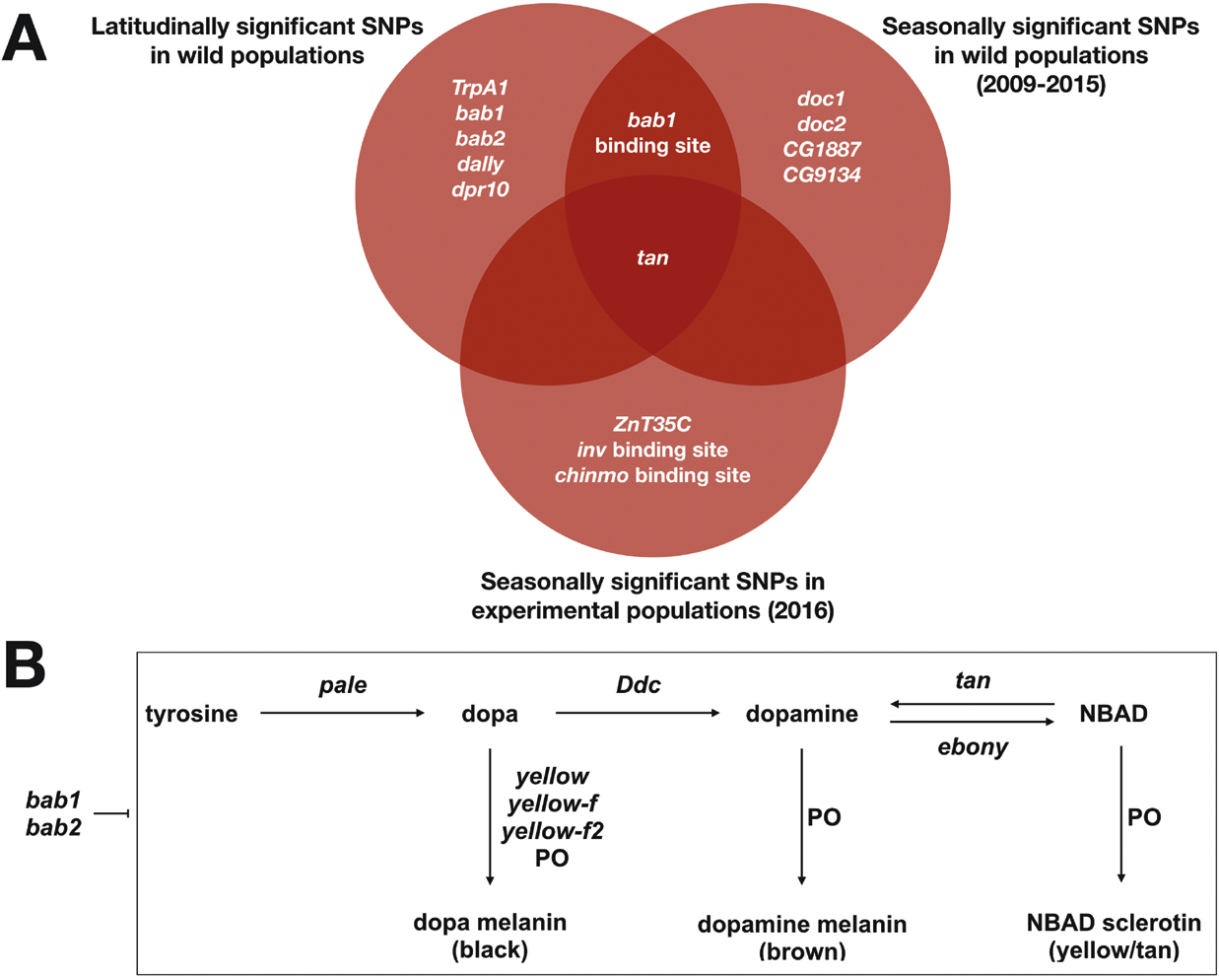
Genes harboring SNPs with significant allele frequency shifts over latitude, seasons in wild populations, and seasons in experimental populations. (A) Diagram of pigmentation genes containing significant SNPs from populations in each natural context. Significance of candidate pigmentation SNPs was determined relative to matched control SNPs, and concordant genes across contexts are displayed. The directionality of allele frequency shifts was co-gradient with observed phenotypic patterns for some SNPs, while other SNPs had countergradient allele frequency shifts. (B) Simplified biosynthetic pathway displaying the canonical pigmentation genes, as well as the relationship between *bab1* and *bab2* to the pathway. *Bab* modulates the pathway by repressing the genes *tan* and *yellow* (Roeske et al., 2018; Salomone et al., 2013), as well as *Ddc* (Gibert et al., 2007).

We assessed whether each pigmentation SNP exhibited a significant allele frequency shift across space or time relative to its matched control SNPs (Supplementary Tables S5A–C and S6). We first identified SNPs that exhibited latitudinal clinality, and they were located in *tan*, *bab1*, *bab2*, a *bab1* binding site (“TFBS_bab1_000953”; modENCODE Consortium, 2010), *TrpA1*, *dally*, and *dpr10* (Figure 4; Supplementary Figure S1; Supplementary Table S5A). We next examined seasonally evolving loci in the populations sampled from our focal orchard in Pennsylvania, cataloging a set of SNPs that (1) evolved rapidly over the course of ~15 generations, and (2) did so in a recurrent manner across 6 years. We observed significant, repeated allele frequency shifts between early- and late-season flies at several loci, including sites within or nearby *tan*, a *bab1* binding site (“TFBS_bab1_000953”), *Doc1*, *Doc2*, *CG1887*, and *CG9134* (Figure 4; Supplementary Figure S2; Supplementary Table S5B). Finally, we analyzed genomic patterns in our experimental orchard. Here, we examined SNPs that exhibited parallel shifts across all replicated populations, and our experimental design eliminated demographic confounds that may be present in the wild populations. The majority of pigmentation SNPs began at a low or intermediate starting frequency (Supplementary Figure S3), and we identified a small set of loci that exhibited detectable allele frequency shifts over time. These SNPs rapidly changed in parallel across all nine mesocosms in fewer than 15 generations, and they included *tan*, *ZnT35C*, and possible binding sites for *inv* and *chinmo* (Figure 4; Supplementary Figure S4; Supplementary Table S5C). Altogether, our analyses uncovered significant allele frequency shifts for several SNPs in both major- (i.e., *tan*, *bab1*, *bab2*) and minor-effect pigmentation genes across all three spatiotemporal scales, but the sets of significant loci varied by context (Figure 4). We then tested whether our significant SNPs exhibited correlated allele frequency shifts between the latitudinal, seasonal, and experimental populations. While we found a slight positive association between the latitudinal and seasonal populations (*R* = 0.295, *p* = 0.25; Supplementary Figure S5A), and between the seasonal and experimental populations (*R* = 0.213, *p* = 0.47; Supplementary Figure S5C), these correlations were not significant. We also found that the latitudinal and experimental populations had a nonsignificant, negative correlation (*R* = −0.145, *p* = 0.62; Supplementary Figure S5B). Additionally, some of the significant SNPs in each context showed countergradient allele frequency shifts to phenotypic patterns (Supplementary Figures S1, S2, and S4).

## Discussion

Here, we provide the first demonstration that abdominal pigmentation in *D. melanogaster* adapts rapidly and predictably as a response to differential environmental conditions, and we uncover shifts in known pigmentation genes that are associated with this change in phenotype. We first found that melanization increases with latitude in North America, mirroring latitudinal clines for pigmentation on other continents (Das, 1995; Munjal et al., 1997; Parkash et al., 2008; Rajpurohit et al., 2008a; Telonis-Scott et al., 2011). We then explored seasonal patterns in a wild population and found that flies repeatedly evolved lighter pigmentation from spring to fall over 6 years. Finally, we recapitulated these seasonal shifts in replicated experimental populations in the field, eliminating any effects of demography while maintaining a natural context. The shifts in our experimental populations were equivalent in both magnitude and direction to the patterns we observed in wild populations, and they were established across nine replicate populations in less than fifteen generations. These data indicate that pigmentation is rapidly adapting in our experimental populations, and therefore also suggest that pigmentation is under selection in wild populations as a response to temperature or other ecological factors that covary with latitude and season. Thus, we found that *D. melanogaster* pigmentation demonstrated concordant and highly predictable adaptive patterns across all spatiotemporal scales.

An important caveat to our phenotypic findings is that the effects of *Drosophila* pigmentation on fitness have not been established in natural populations (Kronforst et al., 2012). It is possible that pigmentation may be under indirect selection due to correlations with other traits (Rajpurohit et al., 2016), rather than direct selection on the degree of melanization itself. While our finding that lighter pigmentation consistently covaries with warmer temperatures provides support for the thermal melanism hypothesis (Freoa et al., 2023), our late-season data hint that the selective landscape of pigmentation may be more complex: populations that adapted to become less melanized following the summer months still exhibited lighter coloration after temperatures began to decrease during the fall. Therefore, the relationship between pigmentation and temperature appears to be somewhat nuanced. UV tolerance, desiccation tolerance, cuticle strength, and wound repair have been posited as alternative benefits associated with *Drosophila* pigmentation (Bastide et al., 2014; Rajpurohit et al., 2008b; Subasi et al., 2024), and future work could investigate the connection between melanism and fitness by (1) manipulating putative agents of selection (e.g., temperature) and observing consequent effects on pigmentation, and (2) testing genetic correlations between pigmentation and other fitness-related traits across multiple axes using a quantitative genetics framework.

Our genomic analyses revealed that common variants of major-effect pigmentation genes are associated with shifts in melanization in each spatiotemporal context, including over ecological timescales: these included *tan*, *bab1*, and *bab2* (Figure 4). This finding reflects the genetic basis of pigmentation variation in natural populations across several taxa, which also experience selection at key loci for pigmentation biosynthesis (Anderson et al., 2009; Barrett et al., 2019; Gratten et al., 2007, 2010; Hoekstra et al., 2006; Jones et al., 2018; Manceau et al., 2011; Miller et al., 2007; van’t Hof et al., 2016). However, while we found that canonical pigmentation genes are associated with spatiotemporal phenotypic variation in *D. melanogaster*, the regulation of melanization appears to be more nuanced. We observed that SNPs in numerous minor-effect pigmentation genes outside of the core melanization pathway also shifted significantly across all scales (Figure 4A and B), and a distinct combination of major- and minor-effect alleles were associated with melanization in each context (Figure 4A). We also failed to identify significant patterns for alleles of other canonical genes involved in pigmentation biosynthesis, including *ebony*, which is associated with altitudinal variation for melanization (Pool & Aquadro, 2007). Thus, adaptive pigmentation patterns appear to be determined by the combined effects of many loci that are incorporated in a context-dependent fashion, rather than exclusively by canonical pigmentation genes.

While a nonoverlapping set of SNPs shifted significantly at the latitudinal, seasonal, and experimental scales, we did observe some parallelism at the gene-level: SNPs in *tan* and *bab1* shifted in multiple contexts (Figure 4). However, the magnitude of these shifts over short timescales appears insufficient to fully account for phenotypic evolution (Supplementary Figures S2 and S4). Many of the major-effect *Drosophila* pigmentation genes are highly pleiotropic (Drapeau et al., 2003; Godt et al., 1993; Jacobs, 1985; Kopp et al., 2000; Massey et al., 2019; Shaw et al., 2000; True et al., 2005; Wilson et al., 1976), so the magnitude of allele frequency shifts in canonical genes may have been limited by the degree to which pleiotropic constraints can be alleviated over each timescale (Pavličev & Cheverud, 2015). We also observed that much of the spatial and temporal allelic variation exhibited countergradient patterns, in which allele frequency differences did not match phenotypes observed (Supplementary Figures S1, S2, and S4). Countergradient patterns have been previously reported for genetic polymorphisms in *D. melanogaster* (Durmaz et al., 2019; Lee et al., 2013; Paaby et al., 2014; Yu & Bergland, 2022), contrasting the simple prediction that allele frequency shifts would be concordant with phenotype. Interactions among multiple combinations of alleles can produce redundant phenotypic patterns, and this can generate allele frequency shifts that conflict in directionality with phenotypic clines (Lotterhos, 2023). Additionally, epistatic interactions influence the effects of individual SNPs on quantitative trait variation across genetic backgrounds in *D. melanogaster* (Huang et al., 2012); thus, epistasis may modulate the effects of a given allele on phenotype across contexts (Bernstein et al., 2019; Noble et al., 2017). Finally, it is important to note that our genomic analyses examined known, common variation in pigmentation genes, so we may not have captured patterns of evolution in any novel, noncoding, or rare variants that contribute to phenotype (Tennessen et al., 2012).

In conclusion, our findings illustrate that *D. melanogaster* abdominal pigmentation evolves as a deterministic response to predictable shifts in environmental conditions that vary over both space and time. Yet, while strikingly parallel pigmentation patterns arose across all spatiotemporal scales, the underlying association between genotype and phenotype varied across all datasets. Therefore, we found that adaptive phenotypic patterns are produced in a predictable fashion over all independent axes analyzed, but the genetic basis of complex trait evolution appears context-dependent. This study contributes to our growing understanding of how patterns of genomic and phenotypic evolution integrate in the field, and whether evolution is predictable in natural populations. Thus, our findings will better inform our understanding of the mechanisms through which populations adapt to rapid environmental change.

## Supplementary Material

qraf008_suppl_Supplementary_Table_S1

qraf008_suppl_Supplementary_Table_S11

qraf008_suppl_Supplementary_Material

qraf008_suppl_Supplementary_Table_S12

qraf008_suppl_Supplementary_Table_S3

qraf008_suppl_Supplementary_Table_S6

## Data Availability

Latitudinal and seasonal genomic data can be accessed via the DEST dataset, v.1 (Kapun et al., 2021). Sequencing data for the mesocosm experiment used in this study have been uploaded to the NCBI SRA under BioProject ID PRJNA1141556. Raw data and scripts have been uploaded to the following GitHub repository: https://github.com/skylerberardi/Papers/tree/main/Berardi_2025_Evol.Lett
