# Supplementary material for: Drosophila melanogaster pigmentation demonstrates adaptive phenotypic parallelism over multiple spatiotemporal scales
Source: Evol Lett. 2025 Apr 8;9(4):408–20. doi: 10.1093/evlett/qraf008 (PMC12448190; doi:10.1093/evlett/qraf008)
Supplement: qraf008_suppl_Supplementary_Material [file qraf008_suppl_supplementary_material.pdf]

Supplementary Materials for

Drosophila melanogaster pigmentation demonstrates adaptive phenotypic parallelism over multiple spatiotemporal scales

Skyler Berardi[1]*, Jessica A. Rhodes[1], Mary Catherine Berner, Sharon I. Greenblum, Mark C. Bitter, Emily L. Behrman, Nicolas J. Betancourt, Alan O. Bergland, Dmitri A. Petrov, Subhash Rajpurohit[2] and Paul Schmidt[1,2]*

Corresponding Authors: Skyler Berardi and Paul Schmidt
Emails: berardis@sas.upenn.edu, schmidtp@sas.upenn.edu

[1] S.B. and J.A.R. contributed equally to this work.
[2] S.R. and P.S. contributed equally to this work.

This file includes:

Figures S1 to S5
Tables S2, S4, and S5
Legends for Tables S1, S3, and S6

Other supplementary materials for this manuscript include the following files:

Datasets (Excel files): Table S1A, Table S1B, Table S1C, Table S3, and Table S6

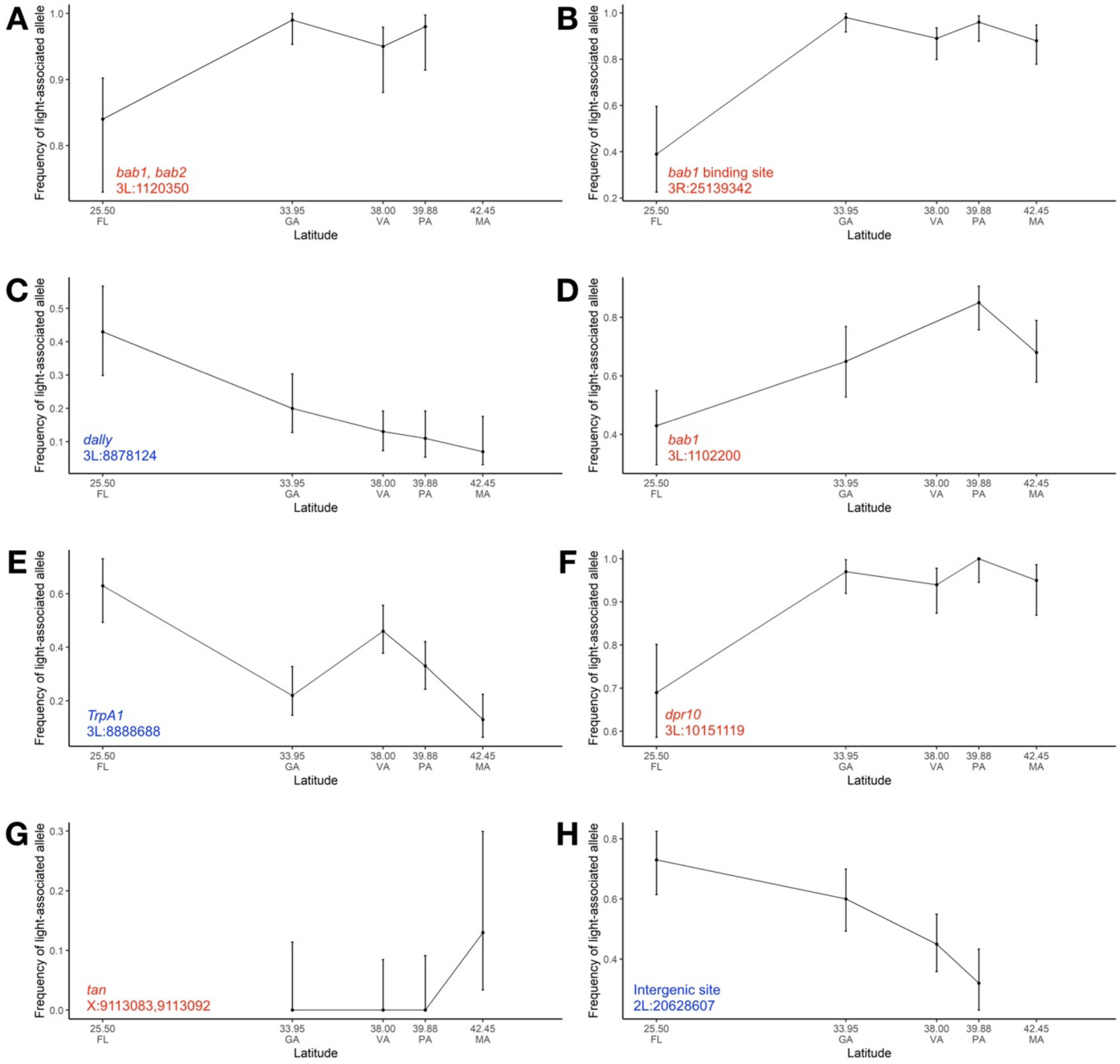

Figure S1. Individual pigmentation SNPs with significant allele frequency shifts across the North American latitudinal cline. Significant SNPs were identified by performing a linear regression of the allele frequencies across latitudes, and then determining if the regression slope for each candidate pigmentation SNP was in the tail of the null distribution created from the slopes of its matched SNPs. The allele frequency of the light-associated allele across latitudes is plotted to illustrate latitudinal differences in SNP frequencies, and error bars represent the 80% confidence interval. Some allele frequency clines were concordant with phenotypic clines (blue text), while other SNPs demonstrated reverse clines (red text).

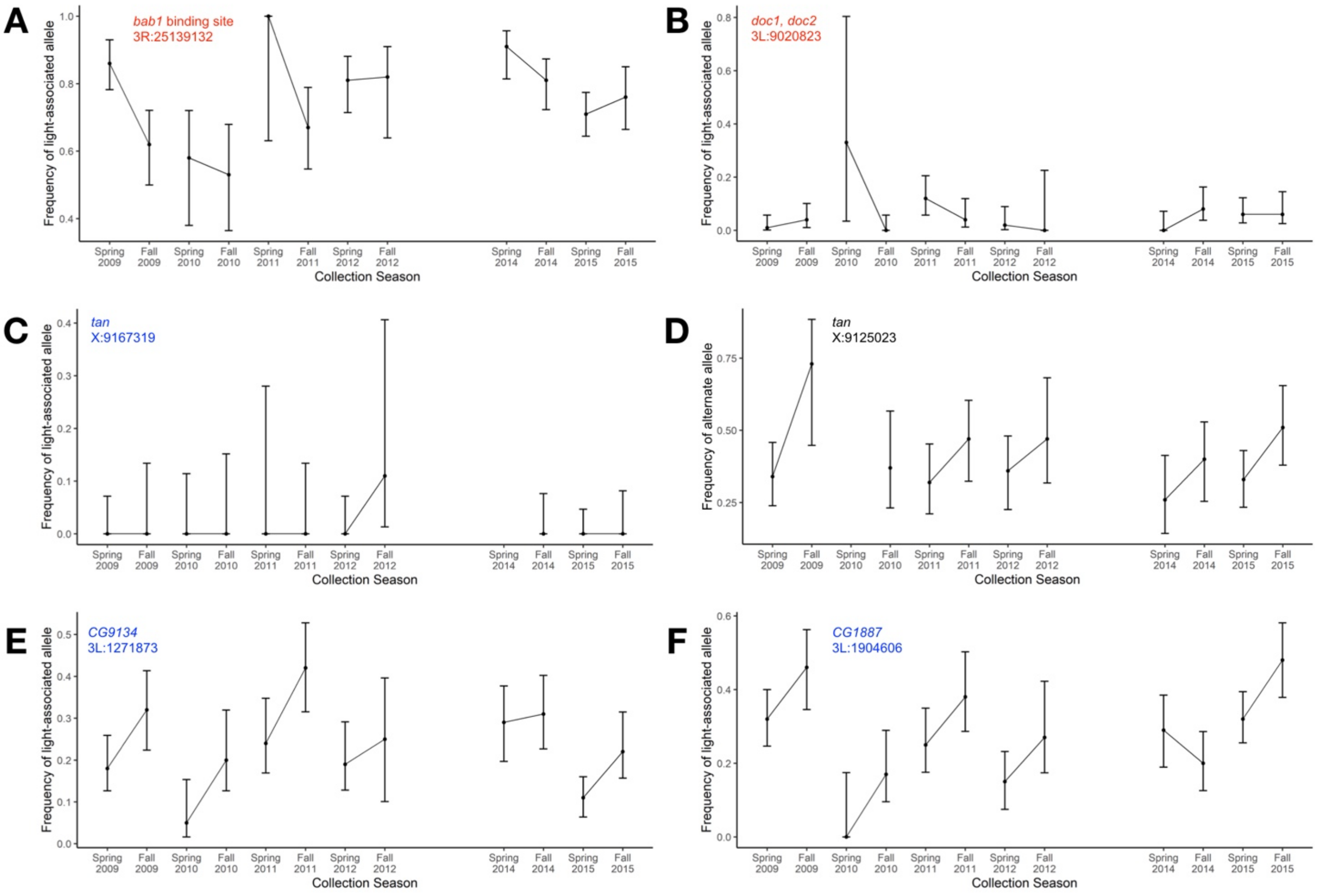

Figure S2. Individual pigmentation SNPs with significant allele frequency fluctuations from spring to fall across multiple years. We determined whether repeated allele frequency shifts were significant by calculating the allele frequency changes from spring to fall in each year and determining the average shift per year for each SNP. We then measured whether the shifts for each candidate pigmentation SNP were in the tail of the null distribution generated from their matched SNPs. The allele frequency of the light-associated allele is plotted across collection seasons to show parallel shifts in SNP frequencies across seasons in multiple years, and error bars represent the 80% confidence interval. Some SNPs exhibited more consistent patterns than others across years, and SNPs showed both co-gradient (blue text) and countergradient (red text) patterns with the phenotypic patterns observed. The directionality for one of the *tan* SNPs (X:9125023) was unspecified in the Bastide et al. (2013) dataset (black text).

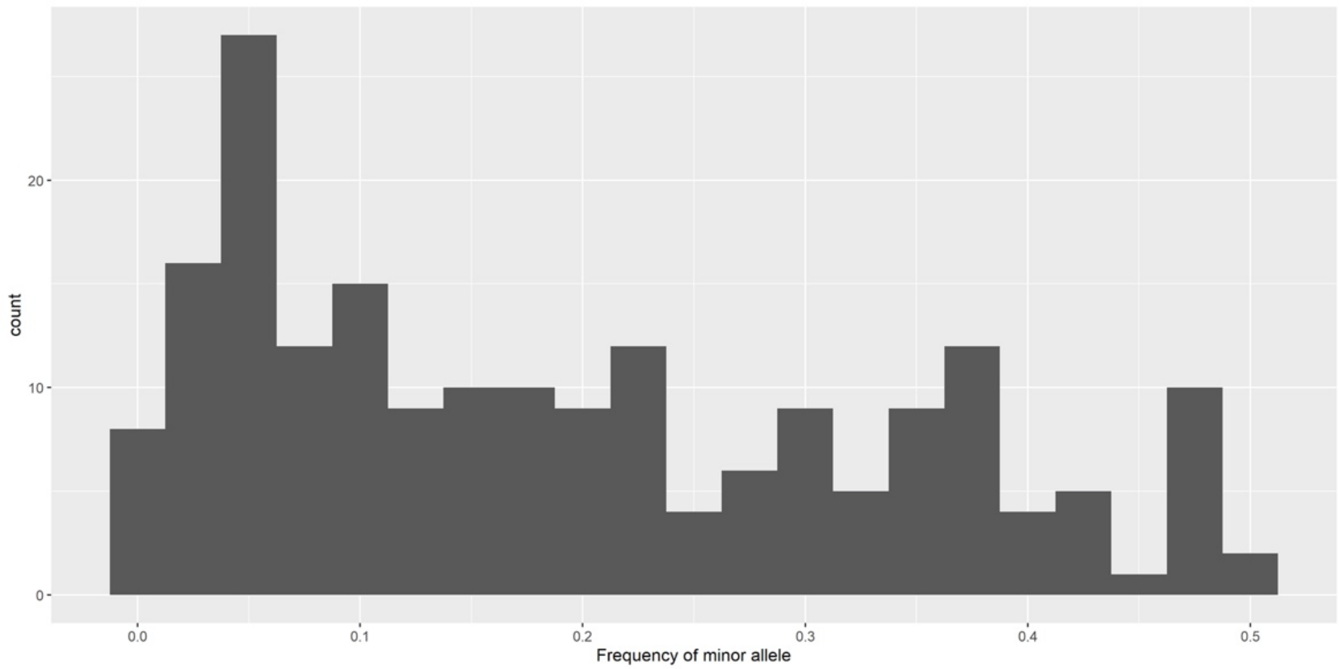

Figure S3. Folded site frequency spectrum displaying the starting frequency of pigmentation SNP alternate alleles in the experimental orchard. The majority of pigmentation SNPs had low or intermediate minor allele frequencies in the baseline population for the experimental orchard, with the largest proportion of initial frequencies falling between 0 and 0.1.

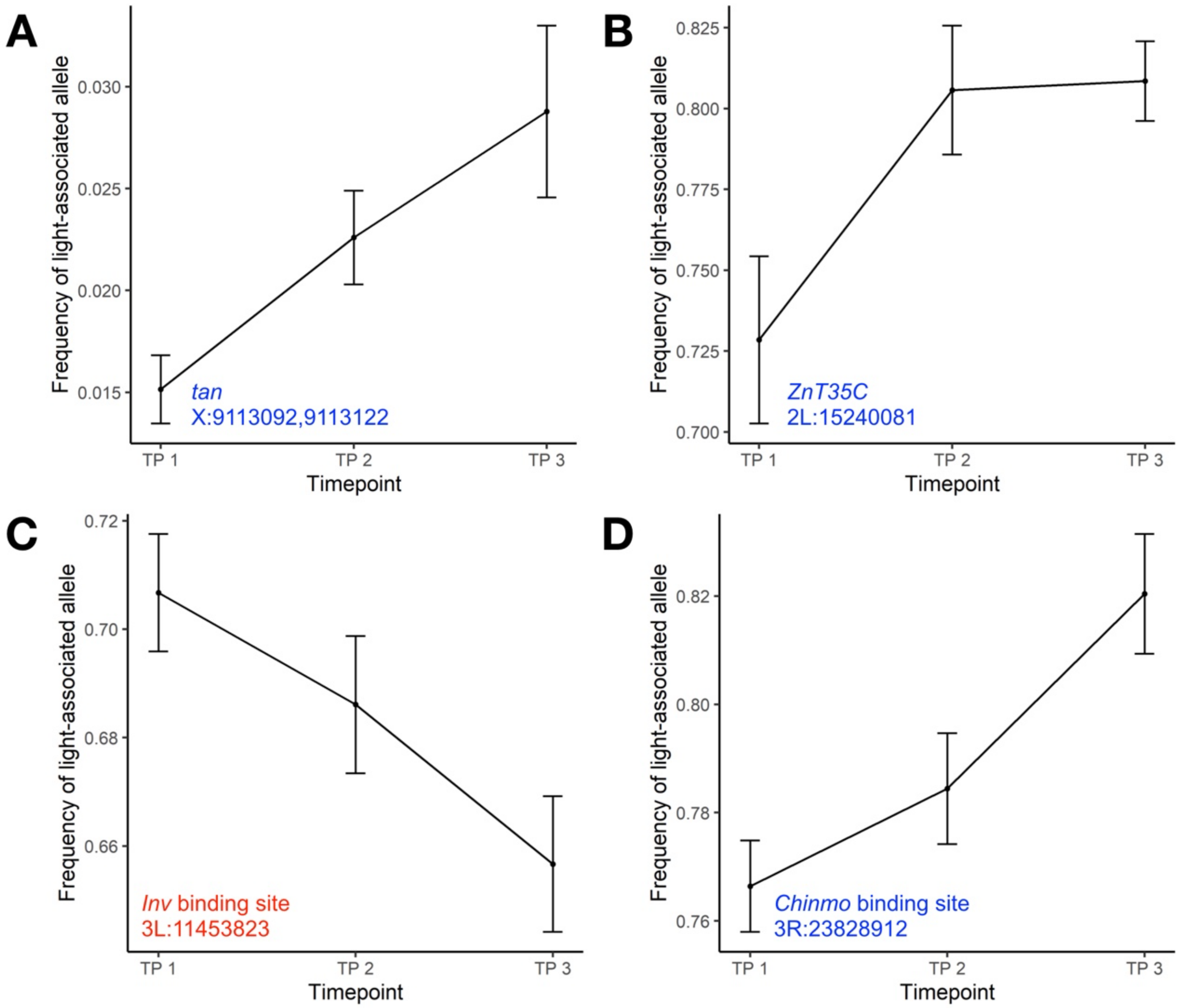

Figure S4. Individual pigmentation SNPs with significant allele frequency shifts across seasons in replicate experimental orchard populations. Significance was determined by first performing a linear regression of the allele frequencies across timepoints, and then identifying if the regression slope for the candidate SNP was in the tail of the null distribution created from the slopes of the matched SNPs. The allele frequency and standard error of the light-associated allele is plotted here to show the movement of SNP frequencies over time. The directionality of allele frequency shifts was concordant with observed phenotypic patterns for some SNPs (blue text), while other SNPs had countergradient allele frequency shifts (red text). (N=10 cages. TP1=Aug. 5, TP2=Sept. 5, TP3=Oct. 5).

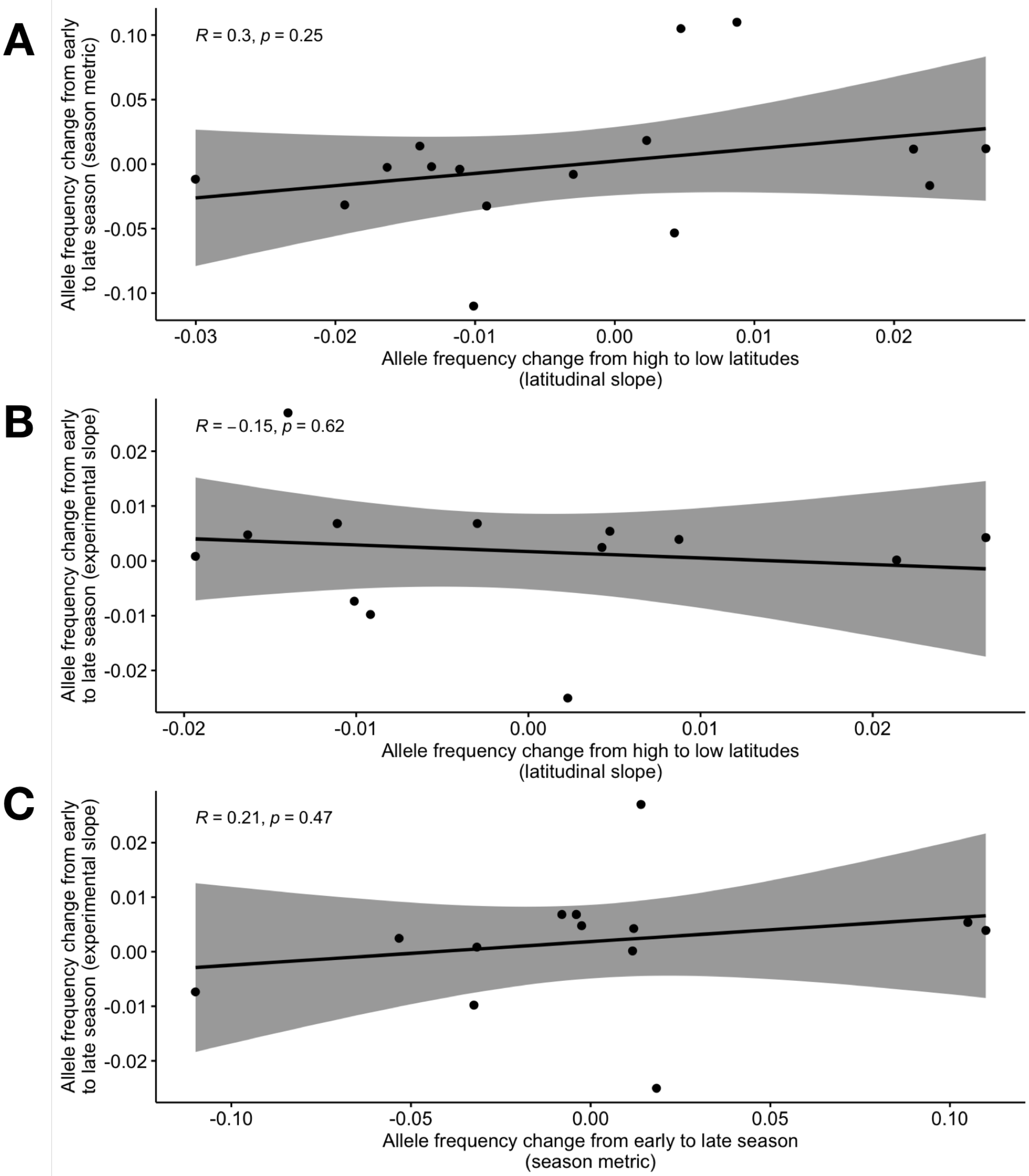

Figure S5. Correlations of allele frequency change for all significant SNPs. We compiled a list of SNPs that exhibited significant change in at least one of the three contexts examined: across latitudinal, seasonal, or experimental populations. We then correlated allele frequency shifts for each SNP between all combinations of contexts. Any SNP that was not present in both populations being examined was excluded for that comparison. Allele frequency change across space or time is represented by the metrics we designed to assess significant shifts in each context: the 'latitudinal slope', 'season metric' and 'experimental slope' (see Methods). Additionally, we polarized all allele frequency metrics to shifts in the light-associated allele. We then used a Pearson's correlation test to assess for significant correlations between the metrics of allele frequency change using the function 'cor.test' in base R. (A) We found a nonsignificant, positive correlation between latitudinal and seasonal allele frequency change ($R = 0.295$, $p = 0.25$). (B) Allele frequency shifts had a nonsignificant, negative correlation between latitudinal and experimental populations ($R = -0.145$, $p = 0.62$). (C) Finally, there was a nonsignificant, positive correlation between seasonal and experimental populations ($R = 0.213$, $p = 0.47$). In all plots, the gray shaded region represents the 95% confidence interval.

Table S1. Pigmentation scoring dataset for (A) latitudinal, (B) seasonal, and (C) experimental populations. Separate Excel files (3) containing raw pigmentation scoring data:
"TableS1A_latitudinal_pigmentation.xlsx"
"TableS1B_seasonal_pigmentation.xlsx"
"TableS1C_experimental_pigmentation.xlsx"

Table S2. Summary table of linear mixed effects model statistics for phenotypic patterns. (A) We determined whether pigmentation varies significantly over latitudes by running a linear mixed effects model using the function lme (R package 'nlme', v.3.1.164). Here, latitude was the fixed effect and isofemale line was included as a random effect. (B) To examine whether pigmentation shifts significantly across seasons in the wild populations (Media, PA), we similarly ran a linear mixed effects model where year, season, and their interaction were fixed effects, and isofemale line was a random effect. (C) Finally, we probed for significant pigmentation patterns across timepoints in our experimental orchard (Philadelphia, PA) by running a linear mixed effects model where timepoint was the fixed effect and mesocosm was a random effect nested within timepoint.

A

Experiment: latitudinal populations
Formula: Pigmentation Scores ~ Latitude, random=~1|Isofemale Line

	numDF	denDF	*F*-value	*p*-value
(Intercept)	1	157	2909.3350	< .0001
Latitude	5	157	11.1707	< .0001

B

Experiment: seasonal wild populations
Formula: Pigmentation Scores ~ Year*Season, random=~1|Isofemale Line

	numDF	denDF	*F*-value	*p*-value
(Intercept)	1	148	5606.354	< .0001
Year	5	148	13.931	< .0001
Season	1	148	174.433	< .0001
Year:Season	5	148	11.517	< .0001

C

Experiment: seasonal experimental populations
Formula: Pigmentation Scores ~ Timepoint, random=~1|Mesocosm/Timepoint

	numDF	denDF	*F*-value	*p*-value
(Intercept)	1	8	2658.3068	< .0001
Timepoint	1	8	111.2574	< .0001

Table S3. Candidate list of pigmentation SNPs from GWAS (Bastide et al. 2013 and Dembeck et al. 2015) included in genomic analyses.
Separate Excel file containing the candidate pigmentation SNP list, "TableS3_pigmentation_SNP_list.xlsx".

Table S4. Testing pigmentation SNP enrichment as a group. Considering all pigmentation SNPs as a group, we compared the distribution of pigmentation SNPs to the distributions of 1,000 matched control SNP groups. We assessed for enrichment of pigmentation SNPs by determining whether they exhibited a significant magnitude of absolute change across spatiotemporal gradients relative to the matched control groups. We completed these analyses using an asymptotic two-sample Kolmogorov-Smirnov (KS) test with a one-sided distribution, and we found that pigmentation SNPs as a group were not enriched in all three contexts.

	D^-	*p-value*	Significant difference between distributions
East Coast Latitudinal Cline Populations	0.037957	0.5233	No
Seasonal Populations (Media, PA)	0.038118	0.5174	No
Seasonal Populations in Experimental Orchard (Philadelphia, PA)	0.022646	0.8231	No

Table S5. Individual pigmentation SNPs with significant allele frequency shifts across each spatiotemporal gradient. (A) List of pigmentation SNPs that exhibited significant allele frequency shifts across latitudinal populations sampled along the East Coast of the U.S. (B) Pigmentation SNPs with significant shifts across seasons in a population sampled from Media, PA, over six years (2009-2012 and 2014-2015). (C) Pigmentation SNPs that shifted significantly across seasons in replicate experimental populations housed in field mesocosms (Philadelphia, PA).

A

HIT	SLOPE	10th percentile of *p*-value distribution	50th percentile of *p*-value distribution	90th percentile of *p*-value distribution	GENE	Concordant with phenotypic directionality?
2L_20628607	0.026569095	0.01	0.02	0.04	intergenic	yes
3L_8888688	-0.022554119	0.009	0.02	0.04	*TrpA1*	yes
3L_1120350	-0.009173534	0.03	0.05	0.081	*bab1, bab2*	no
3L_8878124	-0.021389754	0	0.01	0.021	*dally*	yes
3L_10151119	-0.016300426	0	0.01	0.02	*dpr10*	no
3L_1102200	-0.019337781	0.02	0.05	0.09	*bab1*	no
3R_25139342	-0.030024034	0	0.01	0.01	*bab1* binding site (TFBS_bab1_000953)	no
X_9113092	0.011103075	0	0	0.02	*tan*	no
X_9113083	0.013121816	0	0	0.02	*tan*	no

B

HIT	SEASON METRIC	10th percentile of *p*-value distribution	50th percentile of *p*-value distribution	90th percentile of *p*-value distribution	GENE	Concordant with phenotypic directionality?
3L_9020823	-0.053333333	0.02	0.045	0.07	*Doc1, Doc2*	no
3L_1904606	0.105	0.02	0.04	0.07	*CG1887*	yes
3L_1271873	0.11	0	0.02	0.04	*CG9134*	yes
X_9167319	0.022	0.02	0.04	0.08	*tan*	yes
X_9125023	0.194	0	0.01	0.02	*tan*	*SNP association with phenotype not given*
3R_25139132	0.11	0.029	0.04	0.06	*bab1* binding site (TFBS_bab1_000953)	no

C

HIT	SLOPE	10th percentile of *p*-value distribution	50th percentile of *p*-value distribution	90th percentile of *p*-value distribution	GENE	Concordant with phenotypic directionality?
2L_15240081	-0.040018867	0	0	0.011	*ZnT35C*, binding site for *HSA, sbb*	yes
3L_11453823	-0.025027783	0	0.02	0.03	possible binding site for *inv*	no
X_9113122	0.006818617	0.01	0.03	0.05	*tan*	yes
X_9113092	0.006818617	0.01	0.04	0.061	*tan*	yes
3R_23828912	-0.027007122	0.03	0.05	0.08	possible binding site for *chinmo*	yes

Table S6. Full results of statistical analyses of allele frequency shifts across latitudinal, seasonal, and experimental populations for all individual pigmentation SNPs.

Separate Excel file containing the results of genomic analyses for pigmentation SNP shifts across latitudinal, seasonal, and experimental populations:
"TableS6_pigmentation_SNP_analyses.xlsx"
